# Is the Continued Use of a Small Quantity of Salicylic Acid Detrimental to Health?

**Published:** 1879-08

**Authors:** E. V. Stoddard, H. Kolbe

**Affiliations:** Berlin; Leipsic


### TRANSLATIONS.

---

### "IS THE CONTINUED USE OF A SMALL QUANTITY OF SALICYLIC ACID DETRIMENTAL TO HEALTH?"

TRANSLATED BY E. V. STODDARD, M. D.

In the annual report upon the development of chemical technology, by Rudolph V. Wagner for 1877, p. 451 of the Commercial Report of Gehe, upon the rates of salicylic acid, occurs a note, which runs as follows: "There appears, besides the profit" (in the preservation of Beer by salicylic acid) "not only on the part of the brewer and seller, but also, on the part of the public, that a small quantity of salicylic acid is less injurious to the human organism than the changed products resulting from the acid fermentation of beer, which in common parlance is called 'stich.'" Upon this the editor of the report makes the following observations: "Few of our readers could agree with this opinion. Recent experiences in our own clinic make it decidedly doubtful that salicylic acid could remain indifferent to the compounds of an organism so different from itself."

I give briefly, for evident reasons, my opinion and my own experience on the question, as to whether the continued use of small doses of salicylic acid has any injurious effect upon the health. The clinics, which certainly afford increasing facilities for observation, show that salicylic acid, given to patients in large doses, produces ringing in the ears and other uncomfortable conditions, and also suggests the idea that the continued use of small doses of salicylic acid *may* have the power of producing injurious effects upon the health. This is one of the questions upon which experiment only can justly decide. Such a one have I made upon myself. Since Sept. 5th, I have been daily drinking a watery solution of salicylic acid, containing one gramme (15 gr.) of salicylic acid to one litre (one pint) of water. At first I prepared this solution only; later, I put the same amount in an artificial mineral water, impregnated with carbonic acid; a salicyl-carbonic acid water was the result, which contained one gramme (15 gr.) of salicylic acid to a half litre (½pint) of water, and which was taken diluted with an equal quantity of water. The carbonic acid concealed the taste of the salicylic acid completely of this 1-10 per cent. solution, I daily drink from three-fourths to one litre. I have in this manner, since September, consumed over 200 grammes (6¼ oz.) of salicylic acid. Besides, all the beer and nearly all the wine I have drank during two years past have been salicylized. The excellent draught beer from the Leipsic Brewery, I have regularly drawn from the cask with twenty grammes (300 grains) of salicylic acid to each hektolitre (10 pints) and with wine also ten grammes (150 grains) of salicylic acid to the hektolitre (10 pints).

In this way I have, with water, wine and beer for nine months daily taken at least one gramme (15 grains) of salicylic acid. My health is excellent, I feel better and more vigorous, and am free from the pain which led me to the trial of the salicylic acid as a means of cure. Formerly with the slightest indiscretion in diet, pain in the stomach occurred, and following it, soreness of the mouth and tongue, which frequently rendered speaking painful. During the three-fourths of a year in which I have used the solution of salicylic acid, I have had no recurrence of pain, even after marked errors of diet. The use of salicylic acid has become indispensable to me. It seems from this, to be the same as with spirituous drinks. But few can take half a bottle of rum, while there are but few who do not presume to take a bottle of wine or beer without injury to their health. Hence it may be remarked for the comfort of the habitual beer drinker that he who daily consumes five litres of beer, and, therewith, by an estimate, of 2-10 per cent. solution of salicylic acid, about one gramme of the salicylic acid, in reality perhaps gets the benefit of only the third part of it, while, on the contrary, the full amount of the phosphate of potassa added, would remain in the beer. It is to be remarked that in the use of larger doses of salicylic acid, which would be prescribed in articular rheumatism, the urine contains albumen. My physician, Dr. Bahrdt, of Leipsic, wished to ascertain whether, in the daily use of one gramme (15 grains) of salicylic acid, albumen would be excreted, and, accordingly desired me to examine my urine from time to time. It is always clear, and contains a small quantity of salicylic acid, as may be recognized by the use of the chloride of iron.* Upon

---

* Note. It may be remarked here that the small renal calculi, which previously were often, and not without difficulty, passed off with the urine, during the nine months in which I have regularly drank the solution of salicylic acid have ceased to appear, and accordingly the Carlsbad Course, previously ordered for me by my physician has become unnecessary.

the addition of a few drops of chloride of iron there always appears at once a white precipitate of phosphate of the oxide of iron, more chloride of iron produces the well known blue color. —*From the "Journal für Practische Chemie," from an article by Prof. H. Kolbe of Leipsic.*

Berlin, May 28, 1879.

